# A Novel Predictive Method Incorporating Parameters of Main Pulmonary Artery Bifurcation for Short-Term Prognosis in Non-high-risk Acute Pulmonary Embolism Patients

**DOI:** 10.3389/fphys.2020.00420

**Published:** 2020-04-30

**Authors:** Dong Jia, Xue-Lian Li, Gang Hou, Xiao-Ming Zhou

**Affiliations:** ^1^Department of Emergency, Shengjing Hospital of China Medical University, Shenyang, China; ^2^Department of Epidemiology, School of Public Health, China Medical University, Shenyang, China; ^3^Department of Pulmonary and Critical Care Medicine, First Affiliated Hospital of China Medical University, Shenyang, China; ^4^Department of Pulmonary and Critical Care Medicine, Shengjing Hospital of China Medical University, Shenyang, China

**Keywords:** computed tomography angiography, pulmonary embolism, pulmonary artery, pulmonary hypertension, main pulmonary artery bifurcation

## Abstract

The aim of this study was to build a formula to predict short-term prognosis using main pulmonary artery (MPA) parameters reconstructed from computed tomographic pulmonary angiography in non-high-risk acute pulmonary embolism (PE) patients. After reconstructing the MPA and its centerline, the MPA, the right and left pulmonary artery inlet, and the MPA outlet plane were differentiated to measure the cross-sectional area (CSA), the maximal diameter and the hydraulic diameter. The MPA bifurcation area, volume and angle were measured. MPA dilation was defined as >29 mm at the transverse section plane. The patients were randomly divided into a training set and a validation set. A least absolute shrinkage and selection operator (LASSO) logistic regression algorithm was used to build a predictive formula. The performances of the predictive formula from LASSO were tested by the area under the receiver operating characteristic curve (AUC) and precision-recall (PR) curve with 10-fold cross-validation. The clinical utility was assessed by decision curve analysis (DCA). In total, 296 patients were enrolled and randomly divided (50:50) into a training set and a validation set. The LASSO predictive formula (lambda.1SE) was as follows: 0.92 × MPA bifurcation area + 0.50 × MPA outlet hydraulic diameter + 0.10 × MPA outlet CSA. The AUCs of the predictive formula were 0.860 (95% CI: 0.795–0.912) and 0.943 (95% CI: 0.892–0.975) in the training set and validation set, respectively. The LASSO predictive formula had a higher average area under the PR curve than MPA dilation (0.71 vs. 0.23 in the training set and 0.55 vs. 0.23 in the validation set) and added a net benefit in clinical utility by DCA. Integration of MPA outlet CSA, hydraulic diameter, and bifurcation area with the LASSO predictive formula as a novel weighting method facilitated the prediction of poor short-term prognosis within 30 days after hospital admission in non-high-risk acute PE patients.

## Highlights

- Identifying non-high-risk acute PE patients with a poor short-term prognosis is an effective method to decrease mortality.
- MPA size evaluated by computed tomographic pulmonary angiography is a potential predictor for short-term prognosis, but the predictive ability is limited at the transverse section.
- Further analysis of the MPA size by measuring the MPA outlet hydraulic diameter, the outlet CSA and the main pulmonary bifurcation area could facilitate the prediction of poor short-term prognosis in non-high-risk acute PE patients.
- There were good discrimination abilities after building a predictive formula by the correlative parameters together with MPA dilation in the training set and the validation set (area under the receiver operating characteristic curve: 0.860, 95% CI: 0.795–0912 vs. 0.943, 95% CI: 0.892–0.975; PR curve: 0.71 vs. 0.23 and 0.55 vs. 0.23, respectively). The predictive formula also added a net benefit in clinical utility.

## Introduction

The high-risk group of acute pulmonary artery (PE) patients exhibits greater than 50% mortality with identifiable hypotension characteristics. Reperfusion treatment is essential for treating high-risk acute PE patients (Cok et al., 2013). However, the mortality of non-high-risk acute PE patients who are hemodynamically stable remains at 1–10% in the short term (Becattini et al., 2014). Furthermore, identifying non-high-risk acute PE patients with a poor short-term prognosis is difficult but is an effective method to decrease mortality (Konstantinides et al., 2014).

Currently, there are different strategies for predicting the short prognosis of non-high-risk acute PE patients, such as simplified pulmonary embolism severity (s-PESI), which is useful for the identification of low-risk patients (Lankeit, 2017), and Bova scores, which are reported to miss the diagnosis of some adverse events in short-term follow-up (Fernandez et al., 2015). Computed tomography pulmonary angiography (CTPA) may be a way to improve a prediction. However, the parameters are numerous and miscellaneous. The elevated right ventricular overload caused by pulmonary hypertension (PH) is the main cause of poor short-term prognosis (Lagos-Carvajal et al., 2015). Mechanical obstruction, the release of inflammatory factors and reflex hypoxemia are all involved in the pathophysiology of acute PE (Aviram et al., 2016; Jia et al., 2017). The main pulmonary artery (MPA) cannot react to inflammation and hypoxemia but reflects total PH severity due to its high sensitivity to pressure (Aviram et al., 2011). MPA size evaluated by CTPA (Aviram et al., 2015) is considered a potential predictor of short-term prognosis (Gutte et al., 2017; Beenen et al., 2018). However, there is controversy regarding the short-term prognosis and the MPA size as evaluated by MPA trunk dilation at the transverse section (Seon et al., 2011; Oz et al., 2015; Beenen et al., 2018). At the transverse plane, measurement of the MPA diameter does not represent the MPA size due to tissue compression and good resilience and flexibility of the MPA (Malhotra et al., 2016). Therefore, evaluating only definitional MPA may be insufficient for predicting short-term prognosis. Under PH, the MPA cross-sectional area (CSA), diameters and the sharpness of MPA inevitably change; the MPA bifurcation section is stable and not overly influenced by the cardiac cycle (Schievano et al., 2011), which may be a novel but stable predictor of acute PE short-term prognosis. We propose that evaluating the MPA CSA, hydraulic diameter, maximal diameter and MPA bifurcation size on CTPA may be a superior method of predicting short-term prognosis. To validate this hypothesis, a predictive formula was developed for predicting short-term prognosis at 30 days; internal validation was used to confirm the stabilization of the predictive formula (Moons et al., 2015).

## Materials and Methods

### Study Design and Participants

**FIGURE 1:**
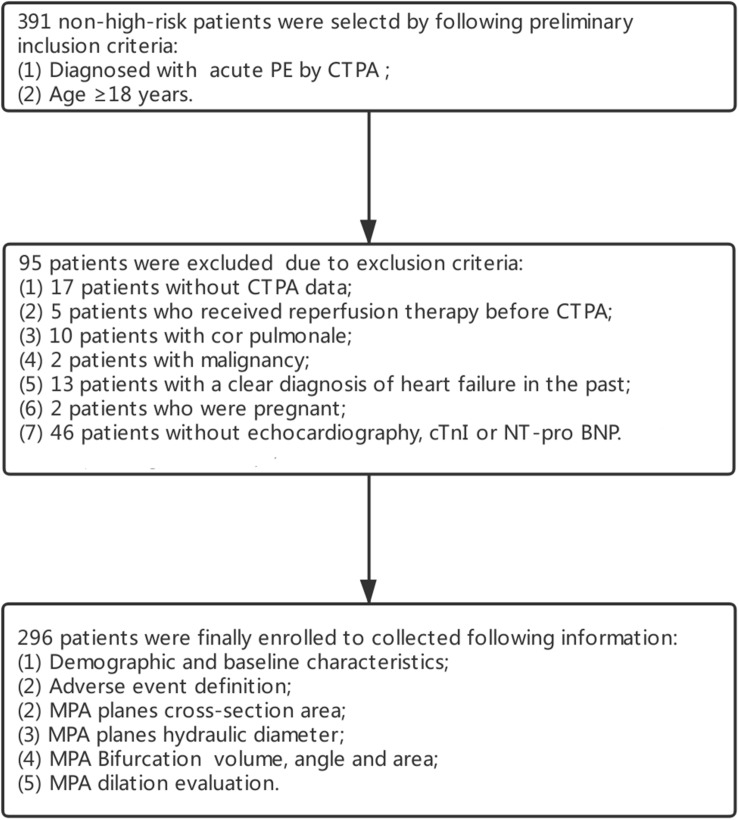
Flow chart of patient inclusion and exclusion.

This retrospective study was conducted at two research centers (Shengjing Hospital of China Medical University and First Hospital of China Medical University) between May 2014 and Oct 2018. Data from 391 non-high-risk acute PE patients (systolic blood pressure ≥90 mmHg, a systolic pressure drop by <40 mmHg and a systolic pressure drop ≥40 mmHg for ≤15 min) (Konstantinides et al., 2014) were initially collected. Patients who were ≥18 years of age and diagnosed with acute PE by CTPA were included. In total, 95 patients were excluded according to the following exclusion criteria: (1) 17 patients lacked CTPA data; (2) 5 patients received reperfusion therapy before CTPA; (3) 10 patients had cor pulmonale; (4) 2 patients had a malignancy; (5) 13 patients had a previous clear diagnosis of heart failure; (6) 2 patients were pregnant; and (7) 46 patients were without echocardiography, cardiac troponin I (cTnI), N-terminal pro-brain natriuretic peptide (NT-pro BNP) or echocardiography. After further screening, 296 patients were ultimately enrolled (see Figure 1).

### Clinical Data and Grouping

**FIGURE 2:**
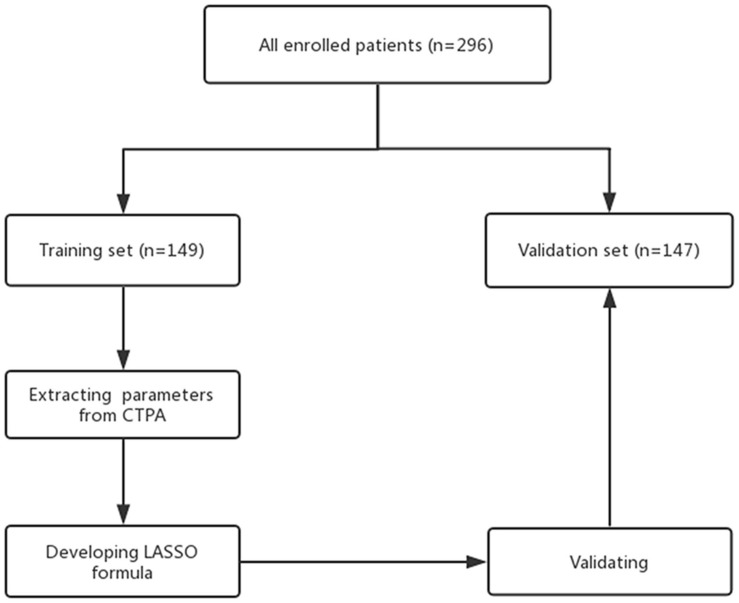
Flow chart of the validation process.

The enrolled patients were randomly divided (50:50) into a training set and a validation set (see Figure 2). To identify a standard short-term prognosis, adverse events were determined within 30 days after admission to the hospital. Adverse events (+) were defined as the occurrence of one of the following events: death; cardiopulmonary resuscitation; endotracheal intubation; vasopressor requirement for systemic hypotension (more than 5 μg per kilogram); or reperfusion treatment to save the patient’s life (Hariharan et al., 2016). Patients without the events listed above were classified as adverse events (−). Adverse events (+)/(−) were used for grouping.

### Risk Stratification

All enrolled patients were divided into the intermediate-high-risk group, intermediate-low-risk group and low-risk group by right ventricular dysfunction (RVD) by echocardiography, cTnI and NT-pro BNP (Konstantinides et al., 2014). cTnI > 0.04 μg/l (normal range 0 ∼ 0.04 μg/l) was defined as cTnI (+); otherwise, patients were classified as cTnI (−) (Binder et al., 2005). NT-pro BNP (+) was defined as NT-pro BNP ≥ 600 pg/ml (normal range ≤300 pg/ml); otherwise, patients were classified as NT-pro BNP (−) (Konstantinides et al., 2014).

### CTPA Acquisition

Computed tomography pulmonary angiography (CTPA) was performed using a 64 detector-row scanner (Aquilion KV-120; Toshiba Medical Systems Corporation). The parameters were as follows: 380 mA; 120 kV; and a 1-mm reconstruction section thickness from the thoracic inlet to the upper abdomen. An iodinated non-ionic contrast agent (100 ml) was injected into the antecubital vein at a rate of 4 ml/s with an automatic dual-tube high-pressure injector (Ulrich REF XD 2051, Ulrich Medical GmbH).

### MPA Parameter Measurements

**FIGURE 3:**
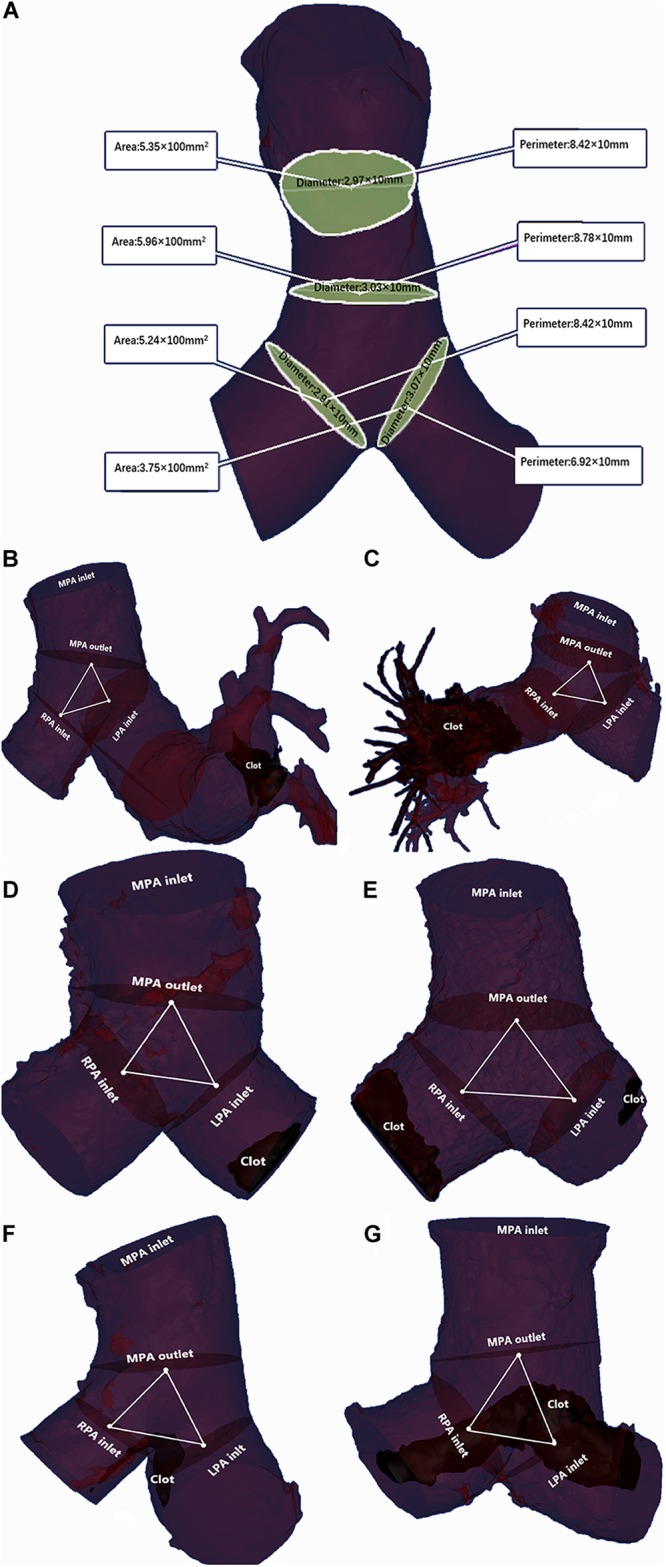
Two examples of MPA parameter measurements. **(A)** The area, perimeter and maximal diameter were measured at the MPA inlet plane, MPA outlet plane, RPA inlet plane and LPA inlet plane. The hydraulic diameter was calculated as the ratio of area to the perimeter of the corresponding plane. **(B)** A 48-year-old female without adverse events and with a clot in the peripheral pulmonary artery: MPA inlet CSA = 6.37 × 100 mm^2^; MPA outlet CSA = 7.68 × 100 mm^2^; LPA inlet CSA = 3.52 × 100 mm^2^; RPA inlet CSA = 3.31 × 100 mm^2^; MPA inlet maximal diameter = 1.97 × 10 mm; MPA outlet maximal diameter = 2.04 × 10 mm; LPA inlet maximal diameter = 3.01 × 10 mm; RPA inlet maximal diameter = 1.62 × 10 mm; MPA inlet hydraulic diameter = 2.86 × 10 mm; MPA outlet hydraulic diameter = 2.94 × 10 mm; LPA inlet hydraulic diameter = 2.04 × 10 mm; RPA inlet hydraulic diameter = 1.97 × 10 mm; MPA bifurcation area = 0.84 × 100 mm^2^; MPA bifurcation angle = 168.35°; MPA bifurcation volume = 9.21 × 1000 mm^3^. The LASSO formula score = 3.01. **(C)** A 59-year-old female with adverse events and a clot in the peripheral pulmonary artery: MPA inlet CSA = 11.79 × 100 mm^2^; MPA outlet CSA = 13.04 × 100 mm^2^; LPA CSA = 6.09 × 100 mm^2^; RPA inlet CSA = 7.56 × 100 mm^2^; MPA inlet maximal diameter = 3.11 × 10 mm; MPA outlet maximal diameter = 3.45 × 10 mm; LPA inlet maximal diameter = 2.48 × 10 mm; RPA inlet maximal diameter = 2.66 × 10 mm; MPA inlet hydraulic diameter = 2.71 × 10 mm; MPA outlet hydraulic diameter = 3.40 × 10 mm; LPA inlet hydraulic diameter = 2.23 × 10 mm; RPA inlet hydraulic diameter = 2.23 × 10 mm; MPA bifurcation area = 5.78 × 100 mm^2^; MPA bifurcation angle = 162.86°; MPA bifurcation volume = 19.53 × 1000 mm^3^. The LASSO formula score = 8.32. **(D)** An 80-year-old female without adverse events and with a clot in the MPA: MPA inlet CSA = 6.72 × 100 mm^2^; MPA outlet CSA = 6.74 × 100 mm^2^; LPA inlet CSA = 4.59 × 100 mm^2^; RPA inlet CSA = 4.63 × 100 mm^2^; MPA inlet maximal diameter = 2.43 × 10 mm; MPA outlet maximal diameter = 2.58 × 10 mm; LPA inlet maximal diameter = 2.06 × 10 mm; RPA inlet maximal diameter = 2.00 × 10 mm; MPA inlet hydraulic diameter = 2.80 × 10 mm; MPA outlet hydraulic diameter = 2.88 × 10 mm; LPA inlet hydraulic diameter = 2.27 × 10 mm; RPA inlet hydraulic diameter = 2.42 × 10 mm; MPA bifurcation area = 0.79 × 100 mm^2^; MPA bifurcation angle = 131.70°; MPA bifurcation volume = 9.69 × 1000 mm^3^. The LASSO formula score = 2.84. **(E)** An 80-year-old female with adverse events and a clot in the MPA: MPA inlet CSA = 12.86 × 100 mm^2^; MPA outlet CSA = 15.04 × 100 mm^2^; LPA CSA = 6.60 × 100 mm^2^; RPA inlet CSA = 6.95 × 100 mm^2^; MPA inlet maximal diameter = 3.76 × 10 mm; MPA outlet maximal diameter = 4.19 × 10 mm; LPA inlet maximal diameter = 2.56 × 10 mm; RPA inlet maximal diameter = 2.85 × 10 mm; MPA inlet hydraulic diameter = 3.67 × 10 mm; MPA outlet hydraulic diameter = 4.15 × 10 mm; LPA inlet hydraulic diameter = 2.80 × 10 mm; RPA inlet hydraulic diameter = 2.77 × 10 mm; MPA bifurcation area = 4.07 × 100 mm^2^; MPA bifurcation angle = 172.35°; MPA bifurcation volume = 40.93 × 1,000 mm^3^. The LASSO formula score = 7.32. **(F)** A 49-year-old male without adverse events and with a saddle clot: MPA inlet CSA = 8.19 × 100 mm^2^; MPA outlet CSA = 9.34 × 100 mm^2^; LPA inlet CSA = 5.64 × 100 mm^2^; RPA inlet CSA = 5.62 × 100 mm^2^; MPA inlet maximal diameter = 2.88 × 10 mm; MPA outlet maximal diameter = 2.86 × 10 mm; LPA inlet maximal diameter = 2.21 × 10 mm; RPA inlet maximal diameter = 2.70 × 10 mm; MPA inlet hydraulic diameter = 2.66 × 10 mm; MPA outlet hydraulic diameter = 2.76 × 10 mm; LPA inlet hydraulic diameter = 2.21 × 10 mm; RPA inlet hydraulic diameter = 2.59 × 10 mm; MPA bifurcation area = 0.88 × 100 mm^2^; MPA bifurcation angle = 165.11°; MPA bifurcation volume = 16.7 × 1000 mm^3^. The LASSO formula scores = 3.12. **(G)** A 69-year-old female with adverse events and a saddle clot: MPA inlet CSA = 85.41 × 100 mm^2^; MPA outlet CSA = 11.72 × 100 mm^2^; LPA CSA = 45.43 × 100 mm^2^; RPA inlet CSA = 46.89 × 100 mm^2^; MPA inlet maximal diameter = 3.17 × 10 mm; MPA outlet maximal diameter = 3.69 × 10 mm; LPA inlet maximal diameter = 2.16 × 10 mm; RPA inlet maximal diameter = 2.40 × 10 mm; MPA inlet hydraulic diameter = 3.11 × 10 mm; MPA outlet hydraulic diameter = 3.45 × 10 mm; LPA inlet hydraulic diameter = 2.31 × 10 mm; RPA inlet hydraulic diameter = 2.52 × 10 mm; MPA bifurcation area = 5.57 × 100 mm^2^; MPA bifurcation angle = 160.06°; MPA bifurcation volume = 13.20 × 1,000 mm^3^. The LASSO formula score = 8.02.

Main pulmonary artery parameters were reconstructed and measured using Mimics Medical software (version 19.0, Mimics Medical software). The detail procedure was shown in Supplementary Videos 1, 2. The centerline was determined based on the reconstructed MPA. Measurement planes were selected perpendicular to the centerline as follows: MPA inlet plane; MPA outlet plane; right pulmonary artery (RPA) inlet plane; and left pulmonary artery (LPA) inlet plane. The method of selecting planes was described by Schievano et al. (2011). At the four selected planes, the CSA, hydraulic diameter and maximal diameter were measured perpendicular to the long-axis of the pulmonary artery (Choi et al., 2015). The hydraulic diameter was measured as 4 × (CSA)/(circumference of the cross section), referring to the study by Louis et al. (2013). In addition, MPA dilation was evaluated by measuring the MPA diameter at the transverse section plane (dichotomized at 29 mm as baseline) and was defined as (+)/(−), referring to the study by Beenen et al. (2018). The MPA bifurcation section, which is defined as the MPA outlet plane and the RPA and LPA inlet planes, is stable across different cardiac cycles (Schievano et al., 2011). The volume of this section was measured and recorded as the MPA bifurcation volume. The area of the three plane linked center points on the centerline was calculated at two dimensions and recorded as the MPA bifurcation area (Das et al., 2015). The angle of the triangle of linked center points toward the MPA was calculated and recorded as the MPA bifurcation angle (see Figures 3A–G).

### Statistical Analysis

Quantitative variables with normal distributions were expressed as the means ± standard deviations (SDs), and differences were analyzed with Student’s *t*-test. Patient sex was expressed as a categorical variable (male/female), and differences were analyzed with the χ^2^ test in a univariate analysis. The histogram was used to exhibit the distribution plots of quantitative variables. First, the sample was randomly divided (50:50). The least absolute shrinkage and selection operator (LASSO) logistic regression algorithm, which is suitable for the regression of high-dimensional data and interactive data analysis (Wu et al., 2017), was conducted by 10-fold cross-validation with penalty parameter tuning based on minimum criteria and 1 standard error of the minimum criteria (the 1-SE criteria) in the training set. With the LASSO method, coefficients of unimportant variables are dropped to zero, while important variables are retained to reduce the overfitting (Zhang and Hong, 2017). A formula with scores as the predictability was developed using selected features that were weighted by their respective LASSO coefficients to predict the endpoint in the training set. The predictive ability of the formula was also calculated in the validation set. The receiver operating characteristic (ROC) curve analysis was conducted to further evaluate the predictive performance of the formula score for predicting the adverse events of the non-high-risk PE patients and for determining the optimal cut-off based on the Youden index (Hanley and McNeil, 1982). Performance abilities of the models for predicting adverse events were compared by calculating the area under the ROC curve (ROC-AUC). The difference in predictive ability between the predictive formula and MPA dilation based on the ROC-AUC was determined using the DeLong test (DeLong et al., 1988). Due to the low morbidity of the adverse events in the non-high-risk PE population, a precision-recall (PR) curve with 10-fold cross-validation was also conducted to evaluate the predictability of the model (Feng et al., 2019). Decision curve analysis (DCA) was calculated to evaluate clinical usefulness by quantifying the net benefits in two sets (Wu et al., 2017). A value of *p* < 0.05 was considered significant. The statistical analysis was performed with R software version 3.3.2^1^ and MedCalc statistical software (version 15.8, Belgium).

## Results

### Comparison of Demographic, Baseline Characteristics and Measurement Parameters in Non-high-risk Acute PE Patients

In total, 296 patients were enrolled after screening. The average age was 60.55 ± 14.46 years. A total of 34 patients (14 males/20 females) were considered adverse events (+) and were grouped into the adverse event (+) group, while 262 patients (118 males/144 females) were considered adverse events (−) and were grouped into the adverse events (−) group. The average ages of participants in the adverse events (+) and (−) groups were 59.23 ± 13.71 and 60.71 ± 14.57 years, respectively. MPA inlet CSA, outlet CSA, inlet hydraulic diameter, outlet hydraulic diameter, RPA inlet CSA, LPA inlet CSA, RPA inlet hydraulic diameter, LPA inlet hydraulic diameter, and MPA bifurcation area and volume in the adverse events (+) group were significantly higher than those in the adverse events (−) group (*p* < 0.001). The MPA bifurcation angle of patients in the adverse events (+) group was significantly higher than that of patients in the adverse events (−) group (*p* = 0.004). The ratio of MPA dilation (+), RVD (+), NT-pro BNP (+), and cTnI (+) in the adverse events (+) group were all higher than those in the adverse events (−) group (all, *p* < 0.001) (Table 1 and Supplementary Figure S1).

**TABLE 1:** Comparison of parameters between adverse events (+) patients and adverse events (−) patients.

**Parameter**	**Adverse events (+) (*n* = 34)**	**Adverse events (−) (*n* = 262)**	***p-*value**
Age (year)	59.32 ± 13.71	60.71 ± 14.57	0.584
Sex (male/female)	14/20	118/144	0.700
MPA inlet CSA (100 mm^2^)	10.43 ± 2.66	7.67 ± 2.15	<0.001
MPA outlet CSA (100 mm^2^)	11.98 ± 3.00	8.39 ± 2.22	<0.001
RPA inlet CSA (100 mm^2^)	6.95 ± 2.89	4.98 ± 1.56	<0.001
LPA inlet CSA (100 mm^2^)	6.71 ± 3.28	4.71 ± 1.41	<0.001
MPA inlet hydraulic diameter (10 mm)	3.28 ± 0.42	2.81 ± 0.39	<0.001
MPA outlet hydraulic diameter (10 mm)	3.50 ± 0.41	2.92 ± 0.42	<0.001
RPA inlet hydraulic diameter (10 mm)	2.60 ± 0.38	2.26 ± 0.35	<0.001
LPA inlet hydraulic diameter (10 mm)	2.39 ± 0.38	2.19 ± 0.32	0.001
MPA inlet maximal diameter (10 mm)	2.90 ± 0.41	2.78 ± 0.37	0.101
MPA outlet maximal diameter (10 mm)	3.24 ± 0.53	3.06 ± 0.44	0.061
RPA inlet maximal diameter (10 mm)	2.23 ± 0.38	2.15 ± 0.37	0.281
LPA inlet maximal diameter (10 mm)	2.15 ± 0.43	2.07 ± 0.38	0.246
MPA bifurcation area (100 mm^2^)	1.32 ± 0.95	0.57 ± 0.31	<0.001
MPA bifurcation angle (°)	133.23 ± 37.04	152.12 ± 22.15	0.004
MPA bifurcation volume (1000 mm^3^)	19.78 ± 8.43	12.90 ± 5.58	<0.001
MPA dilation (+)/(−)	13/21	31/231	<0.001

*MPA, main pulmonary artery; RPA, right pulmonary artery; LPA, left pulmonary artery; CSA, cross-sectional area.*

After randomly grouping the patients, 150 (72 males/78 females) were included in the training set, and 146 (60 males/86 females) were included in the validation set for internal validation. The average ages were 60.25 ± 14.49 years and 60.97 ± 14.46, respectively. There were no differences between the training set and the validation set (Table 2).

**TABLE 2:** Comparison of parameters between the training set and the validation set after random grouping.

**Parameter**	**Training set (*n* = 150)**	**Validation set (*n* = 146)**	***p*-value**
Age (year)	60.25 ± 14.49	60.97 ± 14.46	0.630
Sex (male/female)	72/78	60/86	0.232
MPA inlet CSA (100 mm^2^)	8.12 ± 2.50	7.86 ± 2.25	0.346
MPA outlet CSA (100 mm^2^)	8.97 ± 2.68	8.63 ± 2.61	0.272
RPA inlet CSA (100 mm^2^)	5.30 ± 1.96	5.11 ± 1.77	0.377
LPA inlet CSA (100 mm^2^)	4.94 ± 1.75	4.94 ± 1.92	0.973
MPA inlet hydraulic diameter (10 mm)	2.89 ± 0.43	2.85 ± 0.41	0.329
MPA outlet hydraulic diameter (10 mm)	3.00 ± 0.45	2.97 ± 0.47	0.575
RPA inlet hydraulic diameter (10 mm)	2.31 ± 0.36	2.29 ± 0.39	0.648
LPA inlet hydraulic diameter (10 mm)	2.20 ± 0.33	2.23 ± 0.34	0.458
MPA inlet maximal diameter (10 mm)	2.82 ± 0.39	2.77 ± 0.36	0.278
MPA outlet maximal diameter (10 mm)	3.12 ± 0.47	3.04 ± 0.43	0.135
RPA inlet maximal diameter (10 mm)	2.19 ± 0.40	2.14 ± 0.35	0.269
LPA inlet maximal diameter (10 mm)	2.09 ± 0.40	2.14 ± 0.36	0.546
MPA bifurcation area (100 mm^2^)	0.70 ± 0.52	0.61 ± 0.46	0.141
MPA bifurcation angle (°)	148.55 ± 27.48	151.38 ± 22.12	0.849
MPA bifurcation volume (1000 mm^3^)	13.76 ± 5.70	13.62 ± 6.97	0.329
MPA dilation (+)/(−)	26/124	18/128	0.226
Adverse events (+)/(−)	21/129	13/133	0.169

*MPA, main pulmonary artery; RPA, right pulmonary artery; LPA, left pulmonary artery; CSA, cross-sectional area.*

### Risk Stratification in All Enrolled Patients

A total of 56 patients (25 males/31 females) were sorted into the intermediate-high-risk group, 83 patients (33 males/50 females) were sorted into the intermediate-low-risk group, and 153 patients (74 males and 83 females) were included in the low-risk group. The average ages were 61.46 ± 12.91, 61.47 ± 13.67, and 59.70 ± 15.39 years, respectively (Table 3).

**TABLE 3:** Risk stratification groups for all enrolled patients.

**Parameter**	**Intermediate-high-risk group (*n* = 56)**	**Intermediate-low-risk group (*n* = 83)**	**Low-risk group (*n* = 157)**
Age (year)	61.46 ± 12.91	61.47 ± 13.67	59.75 ± 15.39
Sex (male/female)	25/31	33/50	74/83
Adverse events (+)/(−)	18/38	13/70	3/154

### Comparison of Parameters in the Training Set

A total of 150 patients were included in the training set. A total of 21 (9 males/12 females) patients experienced adverse events (+), and 129 (63 males/66 females) patients did not experience adverse events (−). The average ages were 59.52 ± 11.54 and 60.26 ± 14.95 years, respectively (Table 4).

**TABLE 4:** Comparison of parameters between adverse events (+) patients and adverse events (−) patients in the training set.

**Parameter**	**Adverse events (+) (*n* = 21)**	**Adverse events (−) (*n* = 129)**	***p-*value**
Age (year)	59.52 ± 11.54	60.26 ± 14.95	0.798
Sex (male/female)	9/12	63/66	0.133
MPA inlet CSA (100 mm^2^)	10.38 ± 3.07	7.75 ± 2.20	0.001
MPA outlet CSA (100 mm^2^)	11.84 ± 3.47	8.51 ± 2.22	<0.001
RPA inlet CSA (100 mm^2^)	6.75 ± 3.23	5.06 ± 1.56	<0.001
LPA inlet CSA (100 mm^2^)	6.21 ± 3.13	4.73 ± 1.31	<0.001
MPA inlet hydraulic diameter (10 mm)	3.24 ± 0.40	2.83 ± 0.40	<0.001
MPA outlet hydraulic diameter (10 mm)	3.46 ± 0.44	2.93 ± 0.40	<0.001
RPA inlet hydraulic diameter (10 mm)	2.53 ± 0.36	2.27 ± 0.34	0.004
LPA inlet hydraulic diameter (10 mm)	2.30 ± 0.35	2.19 ± 0.33	0.174
MPA inlet maximal diameter (10 mm)	2.95 ± 0.43	2.80 ± 0.38	0.124
MPA outlet maximal diameter (10 mm)	3.24 ± 0.53	3.06 ± 0.44	0.370
RPA inlet maximal diameter (10 mm)	2.23 ± 0.37	2.18 ± 0.40	0.600
LPA inlet maximal diameter (10 mm)	2.11 ± 0.461	2.09 ± 0.40	0.821
MPA bifurcation area (100 mm^2^)	1.36 ± 1.03	0.56 ± 0.27	<0.001
MPA bifurcation angle (°)	131.42 ± 39.03	151.34 ± 24.19	<0.001
MPA bifurcation volume (1000 mm^3^)	18.04 ± 7.48	13.06 ± 5.06	<0.001
MPA dilation (+)/(−)	8/13	18/111	<0.001

*MPA, main pulmonary artery; RPA, right pulmonary artery; LPA, left pulmonary artery; CSA, cross-sectional area.*

### Formula for Predicting Adverse Events

**FIGURE 4:**
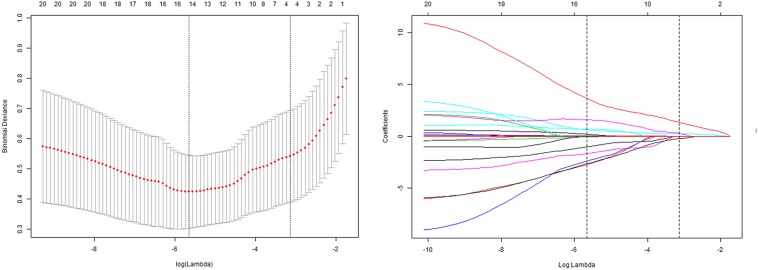
Tuning parameter (lambda) selection in the LASSO model by using 10-fold cross-validation via minimum criteria and 1 standard error of the minimum criteria. Dotted vertical lines were drawn at the optimal values by using the minimum criteria and 1 standard error of the minimum criteria (the 1-SE criteria). The model with the 1-SE criteria was selected. The lambda value of 0.0668, with log (lambda) = –2.70. The model coefficient trendlines of 21 radiomic features; 3 radiomic features were finally included. The formula built by LASSO logistics regression was as follows: score = 0.92 × MPA bifurcation area + 0.50 × MPA outlet hydraulic diameter + 0.10 × MPA outlet CSA.

**FIGURE 5:**
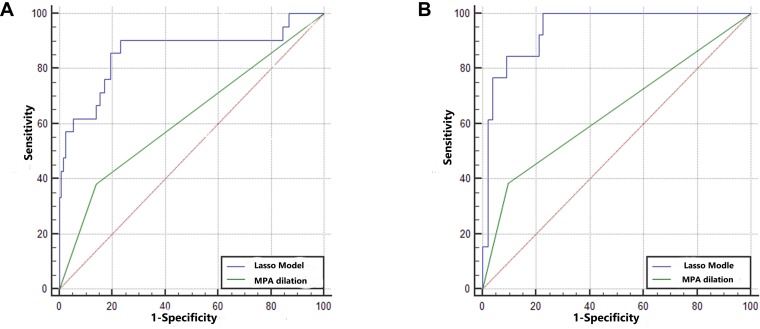
The predictive ability of the training set and validation set. **(A)** The AUCs in the training set were 0.860 (95% CI: 0.795–0.912) and 0.621 (95% CI: 0.538–0.699). The difference between the predictive formula and the MPA dilation measurement was 0.240 (95% CI: 0.115–0.364, *p* < 0.001). **(B)** The AUCs in the validation set were 0.887 (95% CI: 0.892–0.975) and 0.643 (95% CI: 0.560–0.721). The difference between the predictive formula and the MPA dilation measurement was 0.300 (95% CI: 0.164–0.436, *p* < 0.001).

Three radiomic features of MPA parameters with non-zero coefficients were used in the LASSO logistic regression model (the 1-SE criteria) in the training set (see Figure 4). The building of the LASSO mathematical model is shown in Supplementary Figure S2. The formula for calculating the score was as follows: score = 0.92 × MPA bifurcation area + 0.50 × MPA outlet hydraulic diameter + 0.10 × MPA outlet CSA (Figure 4). The ROC-AUC for predicting adverse events in the training set was 0.860 (95% CI: 0.795–0.912, *p* < 0.001). Calculating the score using the formula in the validation set of patients revealed that the ROC-AUC for predicting adverse events was 0.943 (95% CI: 0.892–0.975, *p* < 0.001). There was good discrimination in the training and validation sets (Figures 5A,B).

### Performances of the LASSO Predictive Formula and Clinical Utility in the Training Set and Validation Set

In the training set, the ROC-AUC of MPA dilation, which was measured as the MPA diameter at the transverse section plane, for predicting adverse events was 0.621 (95% CI: 0.538–0.699, *p* < 0.001). The difference between the predictive formula and ROC-AUC of MPA dilation was 0.240 (95% CI: 0.115–0.364, *p* < 0.001). In the validation set, the ROC-AUC of MPA dilation for predicting adverse events was 0.643 (95% CI: 0.560–0.721, *p* < 0.001). The difference between the predictive formula and the ROC-AUC of MPA dilation was 0.300 (95% CI: 0.164–0.436, *p* < 0.001). The predictive ability of the formula was superior to that of the MPA dilation measurement for both the training set and validation set. In addition, for predicting adverse events, the predictive formula was substantially better than the MPA dilation measurement at the transverse section in the PR curve of the training set and validation set (mean precision: 0.71 vs. 0.23 and 0.55 vs. 0.23, respectively) (Supplementary Figure S3). DCA revealed that the predictive formula added more net benefit than the MPA dilation measurement for predicting adverse events in the training set and validation set (range between 0.04 and 1.00 and 0.01 and 0.55, respectively) (Supplementary Figure S4).

## Discussion

Determining ways to use CTPA to predict acute PE prognosis is a popular topic. Evaluating MPA size may be the most effective method for predicting short-term prognosis. However, the predictive ability is still poor, especially at 30 days (Beenen et al., 2018). Further analyzing the MPA morphology in detail to improve identification is essential. By analyzing MPA morphology in detail and building a LASSO predictive formula, we proposed an improved identification method for predicting short-term prognosis. After dividing the patients randomly, an internal validation set was used to confirm the legitimacy of the predictive formula. There were good discriminatory abilities in the training set and the validation set. The technique was also superior to the predictive ability of evaluating only MPA dilation (dichotomizing at 29 mm by MPA trunk diameter).

When PE is triggered, MPA does not dilate as a circle due to good resilience and flexibility and tissue compression. Therefore, measuring only the MPA dilation diameter at the transverse section is not accurate enough. Referring to the deformation and dilation under the increased pressure in airway bifurcation (Liu et al., 2013) and the identical blood flow rate in the MPA bifurcation section (Das et al., 2015), even in cases of PH (Barker et al., 2015), the MPA bifurcation section could be considered an integral section; the shear stress could reflect the pressure in this section. We proposed that measuring the MPA bifurcation angle, area and volume might also be useful for predicting the prognosis of acute PE. We evaluated the area of MPA bifurcation by linking the three center points among MPA bifurcation on the centerline. The elevated right ventricular overload caused by PH was the main cause of poor short-term prognosis (Lagos-Carvajal et al., 2015). This increasing overload can cause the location center point on the MPA outlet plane to shift toward the MPA inlet plane (Schievano et al., 2011), and the location of the centerline on the LPA and RPA shifts toward the two sides. As a result, the MPA bifurcation area based on the centerline increases accordingly. This pathophysiological process caused by PH explains the correlation between adverse events and MPA bifurcation area. In addition, MPA bifurcation angle may not correlate with adverse events due to the trilateral lengthening of the MPA bifurcation triangle simultaneously when PH occurs. The MPA bifurcation volume was also not a predictor of adverse events due to inhomogeneity and the varying morphology of the MPA bifurcation.

After reconstruction, the area and maximal diameter were measured easily by splitting along the centerline (Schievano et al., 2011). The deformation of the MPA was also a non-negligible factor as a reaction to the increasing PH value. Therefore, we proposed that MPA deformation, maximal diameter, CSA and MPA bifurcation size might also correlate with short-term prognosis at four easily identified planes. The results of our study revealed that MPA outlet CSA and hydraulic diameter were correlated to adverse events. The change in MPA CSA was correlative to acute PE prognosis, which represented the efficacy of the pressure on the MPA (Tian et al., 2014). As a tubular structure with high elasticity and low resistance, MPA morphology deforms due to PH. The hydraulic diameter is a parameter for evaluating this deformation by balancing the CSA and perimeter (Louis et al., 2013). CSA and hydraulic diameter were only correlative to adverse events at the outlet plane of the MPA. Less interference and restriction at the MPA outlet plane than at other planes might explain this result (Schievano et al., 2011). Simultaneously, the MPA outlet plane was easier to differentiate than the other planes.

Based on the abovementioned physiopathological considerations, we evaluated MPA morphology in patients in the training set as a potential tool to predict adverse events. MPA outlet CSA, hydraulic diameter and MPA bifurcation area were included in a LASSO predictive formula in the training set, and it showed good discriminatory ability. To confirm its applicability, we continued to evaluate the predictive ability of the formula in the validation set. By bringing the three parameters into the formula and calculating the scores, this predictive formula also showed good discriminatory ability in the validation set. The good discriminatory ability in both the training set and the validation set revealed that the formula was stable. It takes about 5 min to complete all parameter measurements, which is practical in the clinic.

Thus, based on the results of the current study, we employed the more responsive and measurement-stable index of MPA outlet CSA, hydraulic diameter, and bifurcation area on CTPA, which is better than the traditional MPA trunk dilation measurement at the transverse section with better receptivity and less deformation due to the tissue compression and good resilience and flexibility of the MPA, and built a LASSO predictive formula as a novel weighting method. This method provides an independent, practical and rapid prediction, with only three parameters involved in the evaluation, immediately after the definite diagnosis of PE after CTPA. In addition, the clinical benefits associated with the use of this LASSO predictive formula are greater than those of the traditional method.

### Limitations

However, several factors limited the strength of our findings: the retrospective research design limited the strength of the results of our study, as did the small number of adverse events. Choosing internal validation by a random splitting also limited the statistical strength. The formula in our results needs further validation. Our study revealed that the weight ratio of the MPA bifurcation area was the highest in the formula, but it was still not enough to validate the superiority of MPA outlet hydraulic diameter compared with MPA dilation trunk diameter due to the small size of our study.

## Conclusion

Integrating MPA outlet CSA, hydraulic diameter, and bifurcation area with a LASSO predictive formula as a novel weighting method could facilitate the prediction of poor short-term prognosis within 30 days after hospital admission in non-high-risk acute PE patients; this strategy is superior to the use of the MPA dilation diameter in this prediction. Among the three parameters involved, MPA bifurcation area weighed the most markedly.

## Data Availability Statement

The datasets generated for this study are available on request to the corresponding author.

## Ethics Statement

The studies involving human participants were reviewed and approved by Medical Science Research Ethics Committee of the First Affiliated Hospital of China Medical University. Written informed consent for participation was not required for this study in accordance with the national legislation and the institutional requirements.

## Author Contributions

DJ, X-MZ, and GH helped to plan the study, collected the data, and wrote the manuscript. DJ and X-LL helped to analyzed the data. All authors confirmed that they have had full access to data and contributed to drafting of the manuscript.

## Conflict of Interest

The authors declare that the research was conducted in the absence of any commercial or financial relationships that could be construed as a potential conflict of interest.

## Footnotes

1 http://www.R-project.org

## Supplementary Material

FIGURE S1 The histograms of quantitative variables for the exhibition of distribution plots. **(A)** Age; **(B)** MPA inlet maximal diameter; **(C)** MPA inlet CSA; **(D)** MPA inlet hydraulic diameter; **(E)** MPA outlet maximal diameter; **(F)** MPA outlet CSA; **(G)** MPA outlet hydraulic diameter; **(H)** RPA inlet maximal diameter; **(I)** RPA inlet CSA; **(J)** LPA inlet hydraulic diameter; **(K)** MPA bifurcation area; **(L)** MPA bifurcation angle; **(M)** MPA bifurcation volume.

FIGURE S2 The selection of the LASSO mathematical model. Two LASSO predictive models were conducted by 10-fold cross-validation based on minimum criteria (lambda.min criteria) and 1 standard error criteria (the 1-SE criteria) in the training set. **Formula 1** (the 1-SE criteria): Select lambda = lambda.1se: 0.0668 (−2.7065). Variables selected: formula for calculating the score (not including intercept): 0.10 × MPA outlet CSA + 0.92 × MPA bifurcation area + 0.50 × MPA outlet hydraulic diameter, in which three parameters were involved. **Formula 2** (lambda.min) formula for calculating the score (not including intercept): 0.27 × MPA outlet CSA + 1.92 × MPA bifurcation area + 2.01 × MPA outlet hydraulic diameter −0.45 × Left-planet hydraulic diameter −1.17 × MPA outlet maximal diameter − 0.53 × Right-planet hydraulic diameter − 0.44 × LPA inlet maximal diameter, in which severe parameters were involved. The detail procedure for the measurement of the index in **Formula 1 and Formula 2** was shown in Supplementary Videos 1, 2, respectively. Good predictability was achieved in the LASSO mathematical model by each formula. In the training set, the ROC-AUC was 0.897 (95% CI: 0.837–0.941, *p* < 0.05) and 0.860 (95% CI: 0.795–0.912, *p* < 0.05). More parameters (7 parameters) were involved in formula 2, and twice the time as that consumed by formula 1 (3 parameters) was required. Thus, we chose formula 1 (the 1-SE criteria) with the similar predictability but fewer parameters as the prediction method, which might work as a potential and practical tool in clinical practice.

FIGURE S3 The precision-recall (PR) curve of the training set and validation set for predicting the adverse events of the non-high-risk PE patients. The predictive formula was strongly better than that of evaluating the MPA dilation at the transverse section in the PR curve of the training set and validation set for predicting adverse events (mean precision: 0.71 vs. 0.23 and 0.55 vs. 0.23, respectively). **(A)** PR curve in training set; **(B)** PR curve in validation set. Redline is shown to indicate LASSO model; Greenline is shown to indicate MPA dilation. MPA dilation was evaluated by measuring MPA diameter at the transverse section plane (dichotomized at 29 mm as baseline) and was defined as (+)/(−) on CTPA.

FIGURE S4 The decision curve analysis (DCA) showed that utilizing the predictive formula for predicting adverse events added a net benefit both in the training set and in the validation set (range between 0.04 and 1.00 and 0.01 and 0.55, respectively). **(A)** DCA in the training set; **(B)** DCA in the validation set.
