# Supplementary figures and images for: A Novel Predictive Method Incorporating Parameters of Main Pulmonary Artery Bifurcation for Short-Term Prognosis in Non-high-risk Acute Pulmonary Embolism Patients
Source: Front Physiol. 2020 Apr 30;11:420. doi: 10.3389/fphys.2020.00420 (PMC7203501; doi:10.3389/fphys.2020.00420)

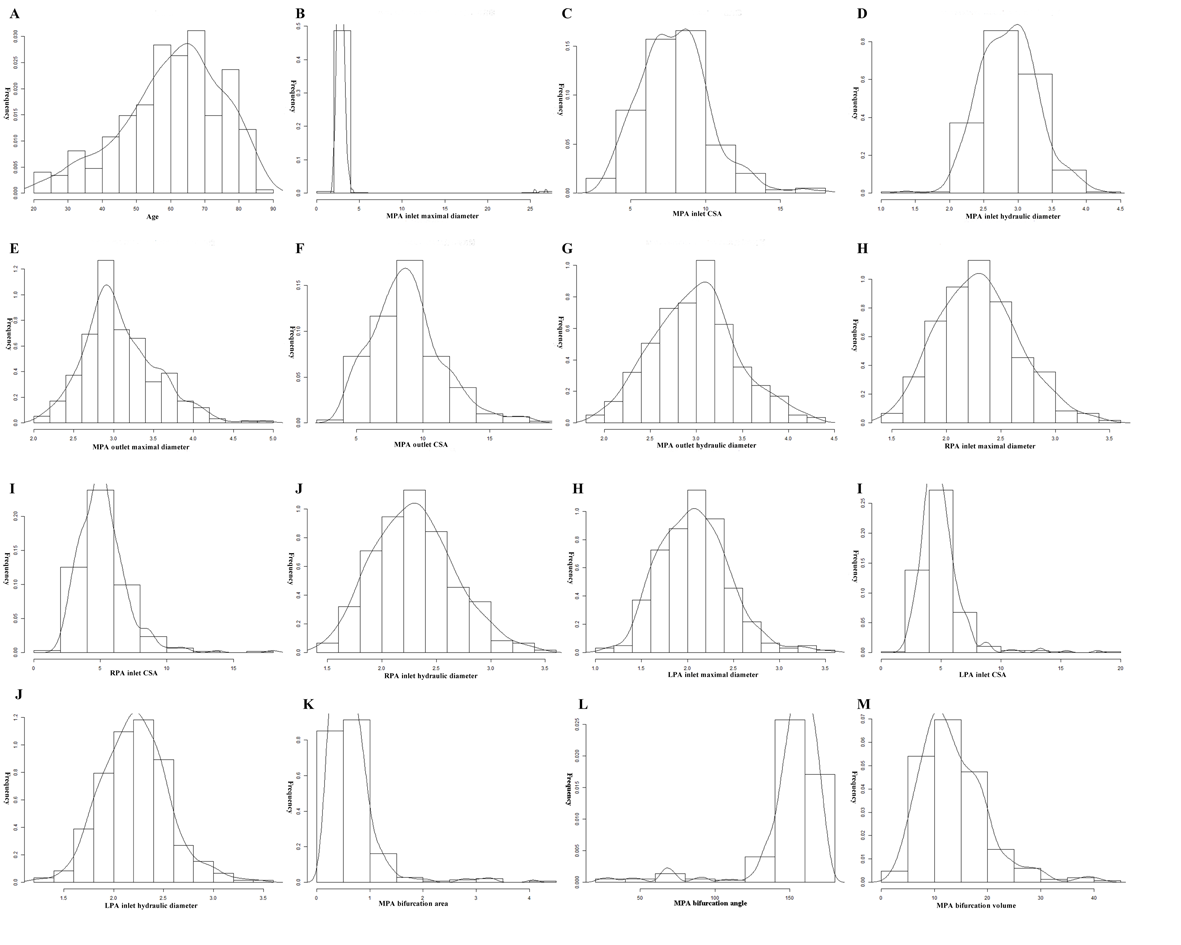

Supplement: FIGURE S1 — The histograms of quantitative variables for the exhibition of distribution plots. (A) Age; (B) MPA inlet maximal diameter; (C) MPA inlet CSA; (D) MPA inlet hydraulic diameter; (E) MPA outlet maximal diameter; (F) MPA outlet CSA; (G) MPA outlet hydraulic diameter; (H) RPA inlet maximal diameter; (I) RPA inlet CSA; (J) LPA inlet hydraulic diameter; (K) MPA bifurcation area; (L) MPA bifurcation angle; (M) MPA bifurcation volume. [file Image_1.tif]

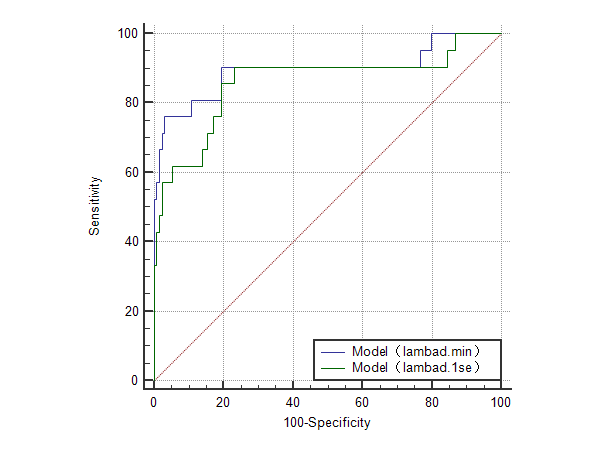

Supplement: FIGURE S2 — The selection of the LASSO mathematical model. Two LASSO predictive models were conducted by 10-fold cross-validation based on minimum criteria (lambda.min criteria) and 1 standard error criteria (the 1-SE criteria) in the training set. Formula 1 (the 1-SE criteria): Select lambda = lambda.1se: 0.0668 (−2.7065). Variables selected: formula for calculating the score (not including intercept): 0.10 × MPA outlet CSA + 0.92 × MPA bifurcation area + 0.50 × MPA outlet hydraulic diameter, in which three parameters were involved. Formula 2 (lambda.min) formula for calculating the score (not including intercept): 0.27 × MPA outlet CSA + 1.92 × MPA bifurcation area + 2.01 × MPA outlet hydraulic diameter −0.45 × Left-planet hydraulic diameter −1.17 × MPA outlet maximal diameter − 0.53 × Right-planet hydraulic diameter − 0.44 × LPA inlet maximal diameter, in which severe parameters were involved. The detail procedure for the measurement of the index in Formula 1 and Formula 2 was shown in Supplementary Videos 1, 2, respectively. Good predictability was achieved in the LASSO mathematical model by each formula. In the training set, the ROC-AUC was 0.897 (95% CI: 0.837–0.941, p < 0.05) and 0.860 (95% CI: 0.795–0.912, p < 0.05). More parameters (7 parameters) were involved in formula 2, and twice the time as that consumed by formula 1 (3 parameters) was required. Thus, we chose formula 1 (the 1-SE criteria) with the similar predictability but fewer parameters as the prediction method, which might work as a potential and practical tool in clinical practice. [file Image_2.tif]

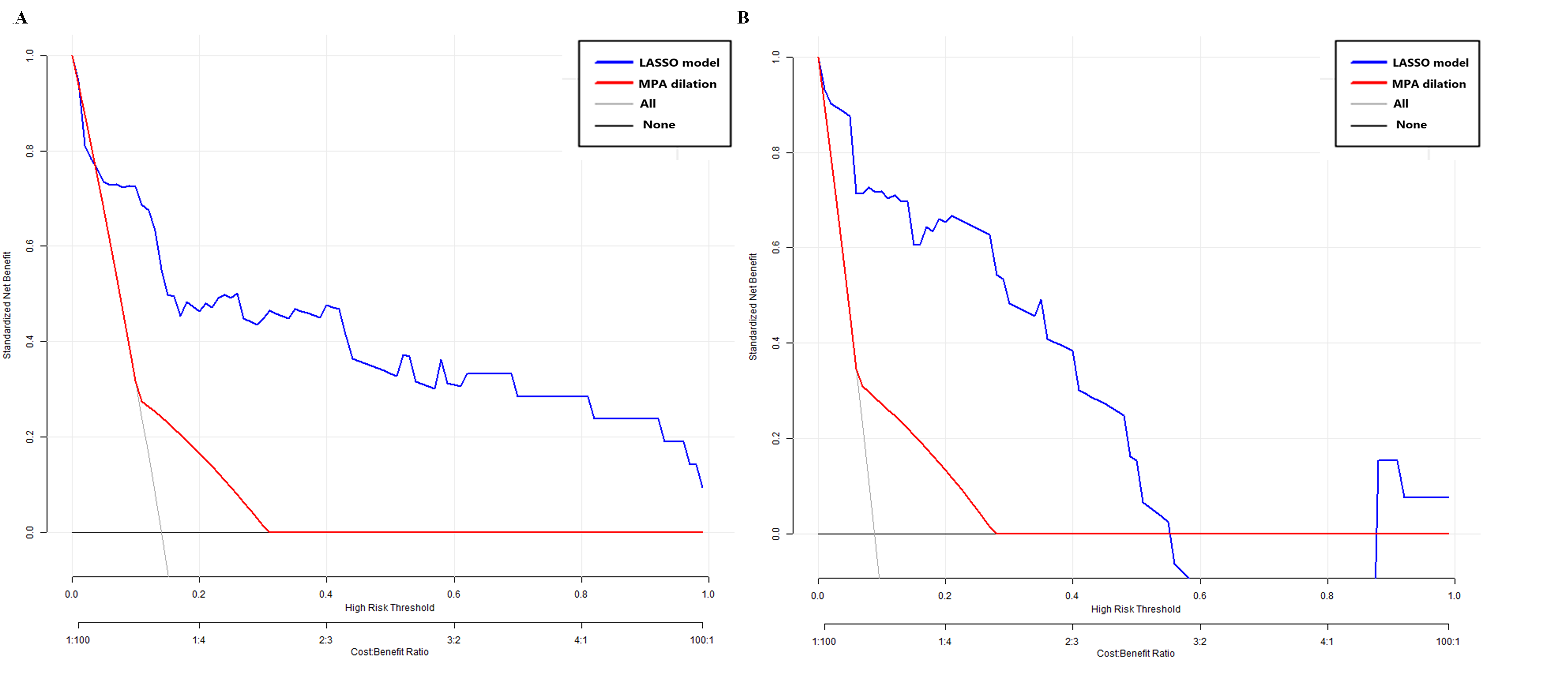

Supplement: FIGURE S3 — The precision-recall (PR) curve of the training set and validation set for predicting the adverse events of the non-high-risk PE patients. The predictive formula was strongly better than that of evaluating the MPA dilation at the transverse section in the PR curve of the training set and validation set for predicting adverse events (mean precision: 0.71 vs. 0.23 and 0.55 vs. 0.23, respectively). (A) PR curve in training set; (B) PR curve in validation set. Redline is shown to indicate LASSO model; Greenline is shown to indicate MPA dilation. MPA dilation was evaluated by measuring MPA diameter at the transverse section plane (dichotomized at 29 mm as baseline) and was defined as (+)/(−) on CTPA. [file Image_3.tif]

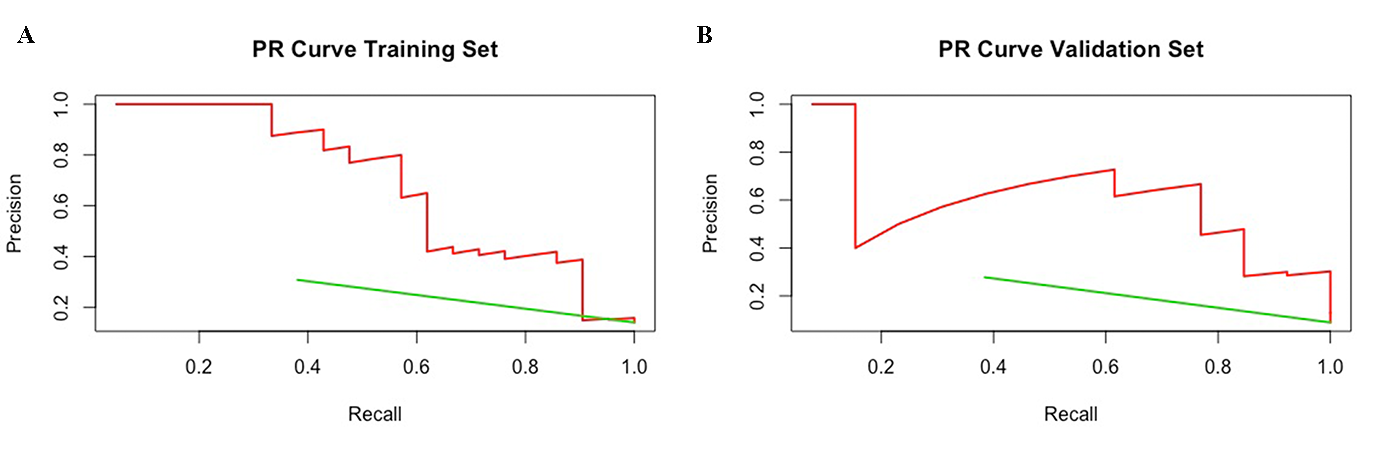

Supplement: FIGURE S4 — The decision curve analysis (DCA) showed that utilizing the predictive formula for predicting adverse events added a net benefit both in the training set and in the validation set (range between 0.04 and 1.00 and 0.01 and 0.55, respectively). (A) DCA in the training set; (B) DCA in the validation set. [file Image_4.tif]
